# Geographic inequalities in paediatric emergency department visits in Ontario and Alberta: a multilevel analysis of 2.5 million visits

**DOI:** 10.1186/s12887-022-03485-x

**Published:** 2022-07-20

**Authors:** Piotr Wilk, Alana Maltby, Tammy Lau, Anna C. Gunz, Alvaro Osornio-Vargas, Shelby S. Yamamoto, Shehzad Ali, Éric Lavigne

**Affiliations:** 1 Department of Epidemiology and Biostatistics, Western University, London, ON Canada; 2 Department of Paediatrics, Western University, London, ON Canada; 3 Child Health Research Institute, London, ON Canada; 4 Lawson Health Research Institute, London, ON Canada; 5 Department of Epidemiology and Biostatistics, Schulich School of Medicine and Dentistry, Western University, 3rd Floor, Western Centre for Public Health and Family Medicine, 1465 Richmond St, ON N6G 2M1 London, Canada; 6 Division of Paediatric Critical Care, Children’s Hospital, London Health Sciences Center, London, ON Canada; 7 Department of Paediatrics, University of Alberta, Edmonton, AB Canada; 8 School of Public Health, University of Alberta, Edmonton, AB Canada; 9 Air Health Science Division, Health Canada, Ottawa, ON Canada; 10 School of Epidemiology and Public Health, University of Ottawa, Ottawa, ON Canada

**Keywords:** Children, Inequalities, Urban, Emergency department, Geographic variation

## Abstract

**Background:**

Research on intra- and inter-regional variations in emergency department (ED) visits among children can provide a better understanding of the patterns of ED utilization and further insight into how contextual features of the urban environment may be associated with these health events. Our objectives were to assess intra-urban and inter-urban variation in paediatric emergency department (PED) visits in census metropolitan areas (CMAs) in Ontario and Alberta, Canada and explore if contextual factors related to material and social deprivation, proximity to healthcare facilities, and supply of family physicians explain this variation.

**Methods:**

A retrospective, population-based analysis of data on PED visits recorded between April 1, 2015 and March 31, 2017 was conducted. Random intercept multilevel regression models were constructed to quantify the intra- (between forward sortation areas [FSAs]) and inter- (between CMAs) variations in the rates of PED visits.

**Results:**

In total, 2,537,442 PED visits were included in the study. The overall crude FSA-level rate of PED visits was 415.4 per 1,000 children population. Across CMAs, the crude rate of PED visits was highest in Thunder Bay, Ontario (771.6) and lowest in Windsor, Ontario (237.2). There was evidence of substantial intra- and inter-urban variation in the rates of PED visits. More socially deprived FSAs, FSAs with decreased proximity to healthcare facilities, and CMAs with a higher rate of family physicians per 1,000 children population had higher rates of PED visits.

**Conclusions:**

The variation in rates of PED visits across CMAs and FSAs cannot be fully accounted for by age and sex distributions, material and social deprivation, proximity to healthcare facilities, or supply of family physicians. There is a need to explore additional contextual factors to better understand why some metropolitan areas have higher rates of PED visits.

## Introduction

According to recent data from the Canadian Institute for Health Information’s (CIHI) National Ambulatory Care Reporting System (NACRS), 21% of ED visits in 2019–2020 occurred among those aged 0 to 19 years in Canada, with 38% of these visits among children 0 to 4 years old [1]. The number of paediatric emergency department (PED) visits in Canada has been increasing [2–4], contributing to healthcare concerns related to ED overcrowding and disruptions to patient flow and care [4]. For instance, in a retrospective cohort study of visits to eight PEDs in four provinces (British Columbia, Alberta, Manitoba, and Ontario), the volume of PED visits increased annually between 2010 and 2014 [3]. For instance, in one PED in British Columbia, the volume of visits increased 30% from 2002 to 2011 [4].

 With approximately 70% of Canadians currently living in census metropolitan areas (CMAs) [5], it is important to understand the determinants of variations in PED visits within (intra-CMA variation) and across (inter-CMA variation) these metropolitan areas [6–8]. These intra- and inter-urban variations may arise from contextual factors such as differential distribution of primary healthcare physicians within and across urban centers [9, 10], or proximity to healthcare facilities [11, 12]. Previous research has indicated that greater supply of primary care providers is associated with fewer non-urgent ED visits among children [13], and proximity to healthcare facilities has also been found to play an important role in the use of EDs [14]. Additionally, some research has highlighted the impact of area-level deprivation on health services utilization as children who live in deprived areas tend to have worse health outcomes than children living in more advantaged areas [6, 15]. With increasing PED visits in Canada, additional research on intra- and inter-regional variations in ED visits among children can provide a better understanding of the patterns of PED utilization and further insight into how contextual features of the urban environment may be associated with these health events.

Currently, there is limited knowledge about geographic inequalities in PED visits in Canada’s urban areas and the contribution of contextual factors to these inequalities, with most studies focusing on intra-urban variation in PED visits for respiratory conditions [6–8]. Therefore, this study aimed to quantify the magnitude of intra-metropolitan and inter-metropolitan variation in PED visits in large urban centers in Ontario and Alberta, Canada and assess if contextual factors related to material and social deprivation, proximity to healthcare facilities, and supply of family physicians can account for this variation.

## Methods

### Study design and setting

A retrospective, population-based analysis of secondary data on ED visits among children (< 18 years old) recorded between April 1, 2015 and March 31, 2017 in the CIHI’s NACRS was conducted for children residing in the provinces of Alberta and Ontario, Canada. Specifically, we selected PED visits recorded in 2015/2016 and 2016/2017 fiscal years to align them with the 2016 Census of Canada. The scope of this study was restricted to CMAs in Alberta and Ontario (19 in total) because only these two provinces had complete ED coverage for all years in the study period [16]. Ontario and Alberta are the largest and fourth-largest provinces in Canada with a population size in 2016 of 13,448,494 and 4,067,175, respectively [17]. Ethical approval was not required as this study used anonymous and confidential secondary data from CIHI. Patient consent was gathered at the time of data collection. All methods were performed in accordance with the relevant guidelines and regulations.

### Study population

After pooling the NACRS datasets together for the two fiscal years, all unscheduled ED visits recorded among patients under the age of 18 residing in CMAs in Ontario and Alberta were included. Duplicate ED records and scheduled ED visits (i.e., when the ED visit date and time was fixed and the appointment was recorded in a scheduling system) were excluded from the analysis, as day surgeries or clinic visits may occur in EDs when organized clinic or day surgery areas are unavailable [18].

### Data source

The NACRS is a population-based health administrative database containing administrative, clinical, and demographic data on emergency and ambulatory care events, which includes ED visits, day surgeries, and outpatient clinic visits [16].

CMAs are geographic areas that are formed by one or more adjacent municipalities centered around a major urban core. A CMA must have a total population of at least 100,000, and at least 50,000 of this population must live in the urban core [19]. To assess intra-metropolitan variation in PED visits, the Forward Sortation Areas (FSA) from patients’ residential addresses were used. FSAs are geographic areas defined by the first three characters of the Canadian postal code and were developed by the Canada Post Corporation to facilitate the delivery of mail [20]. To establish correspondence between FSAs and CMAs, we used Statistics Canada’s Postal Code Conversion File Plus (PCCF +) [21]. As FSAs do not perfectly correspond with Statistics Canada’s census geographic boundaries, a single FSA may be matched with more than one CMA. To account for this, the PCCF + uses a population-weighted allocation matching process [21].

### Measurements

#### Outcome

The outcome of this study was the crude rate of PED visits in each FSA. This rate was calculated by dividing the total number of unscheduled PED visits observed among children residing in each FSA by the FSA-level population under the age of 18 years (derived from the 2016 Census of Canada). The rate was expressed as per 1,000 children population.

#### Contextual predictors

The material and social deprivation indices originally developed by Pampalon et al. from census data were included as measures of FSA-level socioeconomic conditions to assess social inequalities. The indices used in our study were derived from the 2016 census data for the population aged 15 years and older [22, 23]. The material deprivation index is based on the proportion of people without a high school diploma, the employment/population ratio, and average personal income. The social deprivation index is derived from information on the proportion of people living alone, proportion of separated, divorced, or widowed people, and proportion of single-parent families. Statistics Canada’s neighborhood proximity measures were also used to assess the proportion of dissemination blocks in each FSA that have at least one healthcare facility (i.e., physician offices, dentist offices, other health practitioner offices, community health centres, and hospitals) located in its boundaries [24]. Finally, to measure the overall supply of family physicians in each CMA, we used data from the Canadian Medical Association on the total number of family physicians providing services in each CMA [25]. These counts were converted to rates of family physicians per 1,000 population.

#### Confounders

Differences in the distribution of specific demographic characteristics across FSAs may inflate the magnitude of geographic variation in PED visits. To control for some of these compositional effects, we included the following FSA-level factors: the proportion of children in each FSA that was between ages 0–4, 5–9, 10–14, 15–17 years and the proportion of children in each FSA that were female at the time of 2016 census.

### Statistical analysis

To describe the age, sex, and geographic distribution of the PED visits included in our analysis, we produced descriptive statistics (i.e., frequencies and proportions). We also computed means, standard deviations (SD), minimum and maximum values, and interquartile ranges for the outcome variable and the contextual predictors. To quantify the intra- (between CMA specific FSAs) and inter- (between CMA) variations in the rates of PED, three random intercept multilevel regression models were constructed. First, a variance component model (Model 1) with only the outcome variable (FSA-level rate of PED visits) was constructed to estimate the overall magnitude of variation in the rates of PED visits and to partition this variance into FSA- and CMA-level variances. Second, an adjusted model (Model 2) was constructed to control for compositional effects related to age and sex distribution. Finally, we ran a multilevel model in which we tested our hypotheses that FSA-level material deprivation, social deprivation, proximity to healthcare facilities, as well as supply of family physicians in each CMA can account for the observed variation in the rates of PED visits (Model 3). All statistical analyses were conducted using SAS 9.4 (SAS Institute Inc., Cary, NC, USA).

## Results

### Descriptive statistics

Between April 1, 2015 and March 31, 2017, there were a total of 3,352,973 unscheduled PED visits made by children living in Alberta and Ontario. After selecting PED visits made by children residing in one of the 19 CMAs and removing visits from invalid (2,222 visits) and non-residential FSAs occupied by commercial entities (1,558 visits), there were 2,537,442 visits across 520 FSAs (103 FSAs in Alberta and 417 FSAs in Ontario) included in our analysis.

The univariate descriptive statistics of the PED visits are presented in Table 1. There were 1,280,180 PED visits in 2015/2016 and 1,259,262 visits in 2016/2017 fiscal years; overall, 72.4% of the PED visits were from Ontario and 27.6% from Alberta. The average age (years) was 7.17 (SD = 5.66), with the highest proportion of the PED visits made by children aged less than one (10.8%). The sex distribution of PED visits was similar for males (52.9%) and females (47.1%). As indicated in Table 2, the mean crude FSA-level rate of PED visits decreased from 418.0 per 1,000 children population in 2015/2016 to 412.7 in 2016/2017. The lowest rate was 143.6 while the highest rate was 1,131.5 per 1,000 children population. Across both provinces’ CMAs, the crude rate of PED visits was highest in Thunder Bay, Ontario (771.6) and lowest in Windsor, Ontario (237.2).

**Table 1:** Descriptive statistics for paediatric emergency department visits

Individual-Level Factors	Frequency (N)	Percent (%)
*Fiscal year*		
2015/2016	1,280,180	50.5
2016/2017	1,257,262	49.5
*Age*		
0	274,946	10.8
1	275,140	10.8
2	201,261	7.9
3	163,412	6.4
4	159,416	6.3
5	136,102	5.4
6	114,953	4.5
7	103,761	4.1
8	98,409	3.9
9	96,324	3.8
10	96,360	3.8
11	96,786	3.8
12	99,625	3.9
13	104,973	4.1
14	111,910	4.4
15	122,286	4.8
16	133,411	5.3
17	148,367	5.8
*Sex*		
Female	1,194,101	47.1
Male	1,343,199	52.9
Other	142	0.0
*Province of residence*		
Ontario	1,837,555	72.4
Alberta	699,887	27.6

**Table 2:** Descriptive statistics for rates of paediatric emergency department visits and contextual predictors

**FSA-Level Predictors**	**Mean**	**Std Dev**	**Minimum**	**Maximum**	**IQR**
PED visit rate—2015/2016	418.0	165.7	66.7	1,166.3	161.4
PED visit rate—2016/2017	412.7	158.6	142.9	1,109.9	158.6
PED visit rate—2015/2017	415.4	160.6	143.6	1,131.5	156.5
Material deprivation index	-0.01	0.03	-0.11	0.09	0.04
Social deprivation index	0.00	0.03	-0.07	0.09	0.04
Healthcare facility	0.43	0.21	0.02	1.00	0.32
Age 0–4	5.32	1.39	1.69	13.89	1.37
Age 5–9	5.63	1.52	0.76	10.57	1.71
Age 10–14	5.53	1.55	0.47	10.15	1.83
Age 15–17	3.44	0.88	0.33	6.60	1.05
Female	19.09	4.58	3.41	34.72	5.61
Male	20.82	4.89	3.14	36.49	5.81
**CMA-Level Predictors**	**Mean**	**Std Dev**	**Minimum**	**Maximum**	**IQR**
Family physician	1.15	0.26	0.69	2.06	0.35

Rate per 1,000 children population

*CMA* census metropolitan area; *FSA *forward sortation area, *PED* paediatric emergency department, *IQR* interquartile range, *Std Dev* standard deviation

#### Multilevel Regression Models

The results for the random effects from the multilevel regression models are presented in Table 3. In the variance component model (Model 1), the CMA and FSA variance estimates were both statistically significant (16,266 [*p* = 0.0030] and 16,476 [*p* < 0.0001], respectively), with an intraclass correlation coefficient (ICC) of 49.7%. In the adjusted model (Model 2), after accounting for differential distribution of age and sex across FSAs, the CMA and FSA variance estimates decreased by 4.7% and 6.9% but remained statistically significant (15,506 [*p* = 0.0030] and 15,344 [*p* < 0.0001], respectively), with 50.2% of the overall variation in FSA-level PED visit rates attributable to differences across CMAs (i.e., inter-metropolitan variations) and the remaining 49.7% attributable to differences between FSAs (i.e., intra-metropolitan variations).

**Table 3:** Random effects and fit statistics

Model	Estimate	Variance	SE	t-value	p-value	ICC
Model 1	CMA-level	16,266	5,931.11	2.74	0.003	49.68
	FSA-level	16,476	1,041.07	15.83	< .0001	
Model 2	CMA-level	15,506	5,648.62	2.75	0.003	50.26
	FSA-level	15,344	973.48	15.76	< .0001	
Model 3	CMA-level	10,635	4,053.18	2.62	0.0043	45.99
	FSA-level	12,488	794.71	15.71	< .0001	

*CMA* census metropolitan area, *DF* degrees of freedom, *FSA* forward sortation area, *ICC* intraclass correlation coefficient, *SE* standard error

Results for the fixed effects from the final multilevel model (Model 3) indicated that three out of the four contextual predictors (i.e., social deprivation, proximity to healthcare facilities, and supply of family physicians) had a statistically significant association with the FSA-level rate of PED visits; material deprivation was not statistically significant. Specifically, more socially deprived FSAs, FSAs with decreased proximity to healthcare facilities, and CMAs with a higher rate of family physicians per 1,000 children population had higher rates of PED visits; one SD increase in the social deprivation index (SD = 0.03) and in the rate of family physicians (SD = 0.26) as well as one SD decrease in the proximity to healthcare facilities (SD = 21) were associated with an increase in the PED visit rates of 16.7, 44.3, and 68.1 per 1,000 children population, respectively (see Table 4). In terms of the effect of these predictors on the geographic variation, CMA- and FSA-level variances decreased by 31.4% (i.e., from 15,506 to 10,635) and by 18.6% (i.e., from 15,344 to 12,488), respectively (see Table 3).

**Table 4:** Fixed effects

Model	Effect	Estimate	SE	t-value	p-value	Lower	Upper
Model 1	Intercept	467.17	30.57	15.28	< .0001	402.88	531.46
Model 2	Intercept	468.87	29.82	15.72	< .0001	406.15	531.6
	Age 0—4 (REF)						
	Age 5—9	-28.80	17.26	-1.67	0.0959	-62.71	5.12
	Age 10—14	-53.87	15.83	-3.40	0.0007	-84.97	-22.77
	Age 15—17	-1.78	17.22	-0.10	0.9175	-35.62	32.05
	Male (REF)						
	Female	22.40	6.95	3.22	0.0014	8.74	36.06
Model 3	Intercept	445.74	25.00	17.83	< .0001	392.96	498.51
	Age 0—4 (REF)						
	Age 5—9	-1.88	16.11	-0.12	0.9074	-33.53	29.78
	Age 10—14	-18.45	15.93	-1.16	0.2471	-49.74	12.84
	Age 15—17	-12.40	16.71	-0.74	0.4584	-45.24	20.44
	Male (REF)						
	Female	4.54	6.62	0.69	0.4935	-8.47	17.54
	Material deprivation index	-308.56	227.08	-1.36	0.1748	-754.70	137.57
	Social deprivation index	562.82	271.97	2.07	0.0390	28.47	1097.17
	Healthcare facility	-324.41	32.95	-9.85	< .0001	-389.14	-259.68
	Family physician	171.76	75.28	2.28	0.0354	13.13	330.39

*CI* confidence interval, *REF* reference category, *SE* standard error

Finally, Table 5 present (1) crude rates of PED visits for each of the 19 CMAs, (2) estimated rates from the variance component model, (3) estimated rates from the adjusted model, and (4) estimated rates from the adjusted model with four contextual predictors. The results from the model with confounding and contextual predictors indicate that Thunder Bay, St. Catharines – Niagara, Belleville, and Brantford, Ontario had significantly higher rates of PED visits (651.88, 575.59, 563.93, and 553.65, respectively), while Windsor, Ottawa, and Kitchener – Cambridge – Waterloo, Ontario, had significantly lower rates of PED visits (259.09, 341.58, and 358.68, respectively) than the overall mean rate of PED visits of 445.74 per 1,000 children population. None of the three CMAs in Alberta (Calgary, Edmonton, and Lethbridge) had a significantly different rate than the overall rate of PED visits. The results presented in Table 5 indicate that, across all models, the rates in Thunder Bay and St. Catharines – Niagara were significantly higher than the overall rate while the rates for Windsor and Kitchener – Cambridge – Waterloo were significantly lower than the overall rate.

**Table 5:** Census metropolitan area-level rates of paediatric emergency department visits

CMA	Crude Rate	Model 1	Model 2	Model 3
Windsor^*^	237.2	248.3	267.2	259.1
Ottawa^*^	389.2	390.8	395.4	341.6
Kitchener – Cambridge – Waterloo^*^	303.7	310.3	315.4	358.7
Greater Sudbury	395.2	401.2	399.9	369.5
Lethbridge	450.7	453.4	443.4	374.4
Edmonton	469.7	469.6	446.3	395.7
Calgary	457.4	457.6	434.7	405.9
Toronto	348.9	349.6	355.0	406.4
Barrie	381.7	398.9	410.6	415.9
Guelph	376.7	389.8	403.2	416.6
Oshawa	369.6	375.8	389.5	438.9
Average	469.4	467.2	468.9	445.7
London	524.1	521.8	520.3	472.8
Hamilton	474.6	474.3	479.5	476.3
Peterborough	546.0	534.6	532.0	489.8
Kingston	701.5	675.2	680.7	502.5
Brantford^*^	560.5	550.1	561.1	553.7
Belleville^*^	590.4	569.7	565.4	563.9
St. Catharines –Niagara^*^	569.6	564.4	570.8	575.6
Thunder Bay^*^	771.6	740.9	738.4	651.9

^*^p < 0.05

## Discussion

The objective of this study was to assess the intra- and inter-metropolitan variation in PED visits in CMAs in Ontario and Alberta, Canada and to assess if contextual predictors related to material and social deprivation, proximity to healthcare facilities, and supply of family physicians can account for this variation. In total, there were 2,537,442 PED visits across 520 FSAs in CMAs in Alberta and Ontario. The overall crude FSA-level rate of PED visits was 415.38 per 1,000 children population. We found evidence of statistically significant and substantial intra- and inter-urban variation in the rates of PED visits. The interquartile range in FSA-level rates was 156,48 per 1,000 children population and, at the CMA level, the crude rate of PED visits was highest in Thunder Bay (771.65 per 1,000 children population) and lowest in Windsor (237.19 per 1,000 children population). In terms of the intra- and inter-metropolitan variation in PED visits, the FSA- and CMA-level variances were statistically significant across all models (before and after adjusting for age and sex and considering the contextual predictors). About 50% of the overall variance was due to differences between CMAs. The contextual predictors accounted for 31.41% and 18.61% of the CMA- and FSA-level variance, respectively; however, most of the original observed variation was not accounted for. Together, this indicates that there are significant inequalities in FSA-level rates of PED visits across CMAs and across FSAs that cannot be fully explained by age and sex distributions, nor by differences in material and social deprivation, proximity to healthcare facilities, or supply of family physicians. Furthermore, in the adjusted model including the contextual predictors, Thunder Bay, St. Catharines – Niagara, Belleville, and Brantford had significantly higher rates of PED visits than the overall mean rate of ED visits (651.88, 575.59, 563.93, and 553.65 per 1,000 children population, respectively), whereas Windsor, Ottawa, and Kitchener – Cambridge – Waterloo, had significantly lower rates of PED visits (259.09, 341.58, and 358.68 per 1,000 children population, respectively). The three CMAs in Alberta (Calgary, Edmonton, and Lethbridge) were not significantly different from the overall mean rate reported in the adjusted model with contextual predictors.

Our findings indicate that across the 19 CMAs included in this study, Thunder Bay – a CMA in northern Ontario – had the highest average rate of PED visits across all models. This finding aligns with previous research which indicates that residents of northern Ontario have poorer geographic access to primary healthcare, hospitals, and worse health status and outcomes [12, 26]. This is not just limited to adults, as children and youth living in northern Ontario had higher rates of hospitalization and mortality than the provincial rate [27]. Windsor, on the other hand, had the lowest average rate of PED visits across all models. Further research on potential confounding factors is needed to better understand why some children in some CMAs visit EDs more than children in other CMAs.

Geographic inequalities and intra-urban variation have been identified in previous studies in the provinces of Alberta and Ontario. In Calgary and Edmonton, Alberta, Serrano-Lomelin et al. found geographic inequalities and intra-urban variations in use of acute respiratory health services (hospitalizations and ED visits) during early childhood that could not be completely explained by area-level material and social deprivation, suggesting that other unmeasured contextual factors also played a role in influencing the use of these services [7]. They also found that small conglomerate areas across the city of Calgary had greater demand for acute paediatric respiratory health services, whereas in Edmonton, the demand for these services followed a regional-cluster spatial distribution [7]. Sheriff et al. found that the highest number of hot spots for paediatric asthma-related ED visit and re-visit rates in Ottawa, Ontario were within areas that were associated with neighborhood residential instability, material deprivation, dependency, and ethnic concentration [6].

This study addresses inequalities in overall ED utilization among children living in CMAs in Ontario and Alberta. We found that more socially, but not materially, deprived FSAs had increased rates of PED visits. Material and social deprivation have previously been found to be associated with increases in overall [28] and low-acuity [29] ED visits among the general population and with recurrent ED visits among the paediatric population [30]. Belon et al. found that social and material deprivation were significantly associated with episodes of care for paediatric respiratory diseases in Alberta; however, contrary to our study, there was a more consistent gradient of increased rates of ED visits for all respiratory diseases with material deprivation [15]. Children are often accompanied by parents or caregivers to the ED, and parents’ decision to seek care in EDs are complex and often extend beyond measures of socioeconomic status. Driving factors for parents bringing their child to the ED include feelings of anxiety, urgency, need for immediate care and reassurance, perceptions that the ED was the best place to receive care, and the convenience and access of EDs (i.e., not needing appointment and around-the-clock care). These feelings and perceptions may be exacerbated in single-parent families or among those who experience greater social deprivation, resulting in higher rates of ED visits [31, 32].

Closer proximity to healthcare facilities was associated with reduced rates of PED visits in our study, which is consistent with previous research. Results from a study in the United States found that children living closer to their primary care physician had lower ED use, while those living closer to an ED had higher ED use [14]. Although we did not assess proximity to hospitals separately, research has indicated that proximity to an ED results in higher use. In British Columbia, geographic proximity was one of the top reasons for parents’ bringing their child to the ED for non-emergent complaints [33]. Similarly, Shechter et al. found that living closer to the ED compared to the clinic was a significant predictor of ED utilization among children in the United States [34]. Thus, separate measures of proximity to specific healthcare facilities (e.g., hospitals, primary healthcare clinics) should be considered in future studies assessing geographic inequalities in PED visits.

Interestingly, we found that CMAs with a greater supply of family physicians also had higher rates of ED visits. While having greater numbers of family physicians would seemingly reduce rates of ED utilization, our results may stem from the unequal distribution of family physicians in an area, as opposed to absolute supply [9, 13]. Furthermore, the accessibility of family physicians and primary healthcare services (e.g., hours, location) may also contribute to ED visits. Children in the United Kingdom registered in practices that were easily accessible were 9% less likely to visit the ED [35]. Given that the contextual factors related to social and material deprivation, proximity to healthcare facilities, and supply of family physicians did not account for all the variation in PED visits, it is likely that other social and environmental factors at the neighbourhood-level, (e.g., safety, air pollution, and features of the built environment), may also affect the utilization of the ED by children.

### Strengths and limitations

This study is not without limitations. First, FSAs were used to assess inter-metropolitan variation within CMAs because these were the smallest geographic units available; however, FSAs are known to have irregular boundaries and vary in size. Although we believe that FSAs are sufficient to assess overall intra-metropolitan variation, future research is warranted using smaller units of geography as using such units could have generated different results. Secondly, 19 CMAs from Alberta and Ontario were used because only these two provinces are mandated to submit ED data to CIHI. Although studies tend to focus on only a single province, city, or hospital, by assessing both Alberta and Ontario, we provided a better understanding of across CMA variation in PED visits in Canada. For this study, we were also able to link the ED data with contextual factors derived from the census data. Lastly, the health administrative data used in this study poses limitations related to the information included as these data do not capture any exposure related details that may be used to assess variation in PED visits.

## Conclusions

In conclusion, there are geographic inequalities in the rates of PED visits across FSAs in Canada’s large urban centers. As some urban areas have higher rates of PED visits, there is a need to further explore the role of contextual factors, as they are important in addressing health inequalities, particularly in urban settings. It would also be beneficial to extend the scope of the current study to assess spatial distribution of inequalities in PED visits.

## Data Availability

Data is available only through a data sharing agreement between Health Canada and the Canadian Institute for Health Information and the authors are not permitted to share the data (more information on accessing the data can be obtained by contacting help@cihi.ca). The analyses and the interpretation are the authors’ alone.

## Abbreviations

CMA: Census metropolitan area
ED: Emergency department
FSA: Forward sortation area
PED: Paediatric emergency department
